# Psychomotor effect differences between *l*-methamphetamine and *d*-methamphetamine are independent of murine plasma and brain pharmacokinetics profiles

**DOI:** 10.1007/s00702-017-1694-y

**Published:** 2017-02-17

**Authors:** Tetsuya Nishimura, Kazue Takahata, Yuri Kosugi, Takaaki Tanabe, Shizuko Muraoka

**Affiliations:** Department of Scientific Research, Fujimoto Pharmaceutical Corporation, 1-3-40 Nishiotsuka, Matsubara, Osaka 580-8503 Japan

**Keywords:** Methamphetamine, Enantiomer, Pharmacokinetics, Psychomotor

## Abstract

*l*-Methamphetamine has been occasionally referred to as a stimulant similar to *d*-methamphetamine, probably owing to insufficient comparative studies. Here, we directly compared psychomotor efficacies and pharmacokinetics of methamphetamine enantiomers in mice. Only *d*-methamphetamine, but not *l*-methamphetamine, induced stereotypy and sensitization at 1–10 mg/kg. However, plasma pharmacokinetic parameters of 10 mg/kg *l*-methamphetamine were ≥tenfold those of 1 mg/kg *d*-methamphetamine. These results clearly indicate that differential psychomotor efficacies of methamphetamine enantiomers are independent of their pharmacokinetic profiles.

## Introduction

Methamphetamine is a highly addictive stimulant, and its psychostimulant effects have been suggested to be attributable to its stimulating action on presynaptic neurons, resulting in a release of dopamine and other neurotransmitters through monoamine transporters or vesicular monoamine transporters (Barr et al. 2006). Methamphetamine, having a chiral center, exists as *d*- and *l*-enantiomers and is designated as a controlled substance without discrimination of its enantiomers. The *d*-enantiomer exerts potent physiological and psychostimulant effects and has high abuse liability, whereas the *l*-enantiomer exerts almost none of these effects (Mendelson et al. 2006). In clinical practice, *d*-methamphetamine is prescribed for treatment of attention-deficit/hyperactivity disorder, exogenous obesity, and narcolepsy. *l*-Methamphetamine is an active ingredient contained in a nasal decongestant (Vicks Vapor Inhaler) in the United States and is a metabolite of selegiline, a selective monoamine oxidase (MAO)-B inhibitor widely used for treatment of Parkinson’s disease and depression. *l*-Methamphetamine has often been described as a molecule with pharmacological efficacy comparable to *d*-methamphetamine, likely because only a few comparative pharmacodynamic and pharmacokinetic studies have been conducted. Therefore, selegiline, sometimes ambiguously referred to as its major metabolite *l*-methamphetamine, may also induce psychostimulant effects.

The aim of the present study was to determine the efficacies of the methamphetamine enantiomers to induce psychostimulant effects, and to clarify a cause for any differences. Some pharmacological response differences are related to pharmacokinetic properties. For instance, a comparative study on *d*-methamphetamine and cocaine revealed that the slower clearance of *d*-methamphetamine contributes to the longer-lasting stimulant effects (Fowler et al. 2007). Thus, in the present study, we directly compared the psychomotor effects and pharmacokinetics of the methamphetamine enantiomers in mice.

## Materials and methods

### Animals

Male ddY mice (8 weeks old, Japan SLC, Shizuoka, Japan) were kept in a facility with controlled humidity (50 ± 20%) and temperature (23 ± 2 °C) and were maintained under a 12-h light/dark cycle with free access to food (Oriental Yeast, Tokyo, Japan) and water. The mice were acclimated for 1 week before being used in the experiments.

### Chemicals

*l*-Methamphetamine hydrochloride was prepared from benzaldehyde in our institution according to previously described methods (Paulsen-Sörman et al. 1984; Posakony et al. 2002). The purity of the product was >99%. *d*-Methamphetamine hydrochloride was purchased from Dainippon Pharmaceutical (Osaka, Japan). All reagents were dissolved in saline and administered subcutaneously (s.c.).

### Locomotor activity

Locomotor activity was measured for 2 h post-drug administration using an infrared-linked activity sensor system (AB System-24A, Neuroscience, Tokyo). For sensitization, each mouse was treated with one of the enantiomers at an interval of 3 or 4 days, for a total of seven injections. Locomotor activity in these mice was also measured for 2 h post-drug administration.

### Stereotyped behavior

The intensity of stereotyped behavior was assessed at 15-min intervals for 2 h post-drug administration using the scoring system of Costall and Naylor (1973): 0, behavior of the mouse is the same as that of a saline-treated mouse; 1, discontinuous sniffing with constant exploratory activity; 2, continuous sniffing and periodic exploratory activity; 3, continuous sniffing and discontinuous biting, gnawing or licking; 4, continuous biting, gnawing or licking, with no exploratory activity.

### Pharmacokinetics

A blood sample (20 μL) was collected from tail vein at indicated time points in Table 1, and stored at −20 °C after centrifugation (12,000×*g*, 5 min). The striatum was dissected out 2 h after administration and stored at −80 °C. Striatal samples were homogenized in 50% acetonitrile, and centrifuged (10,400×*g*, 15 min, 4 °C). Each sample was extracted with 1-chlolobutane/acetonitrile (4/1, v/v), then with 0.5% HCl (back extraction). Amphetamine and methamphetamine concentrations were determined by liquid chromatography–tandem-mass spectrometry (Slawson et al. 2002) with a Chromolith RP-18e column (Merck, Darmstadt, Germany), without chiral derivatization (Nishida et al. 2006). The lower limit of quantification was 3 ng/mL, but for brain amphetamine, 1 ng/mL. The maximum plasma concentration (*C*_max_) and the area under the plasma concentration vs. time curve from 0 to 2 or 4 h (AUC_0–2h_ or AUC_0–4h_) were calculated using WinNonlin software version 6.4 (Certara, NJ, USA).

### Statistical analysis

Statistical analyses were performed using one-way analysis of variance with SPSS Statistics software (IBM Corp., NY, USA), followed by Dunnett’s test (locomotor activity and stereotypy), the Bonferroni correction (sensitization), or Student’s *t* test (pharmacokinetics). Differences were considered statistically significant at values of *P* < 0.05.

## Results

### Comparison of methamphetamine enantiomer-induced psychomotor effects

**Fig. 1:**
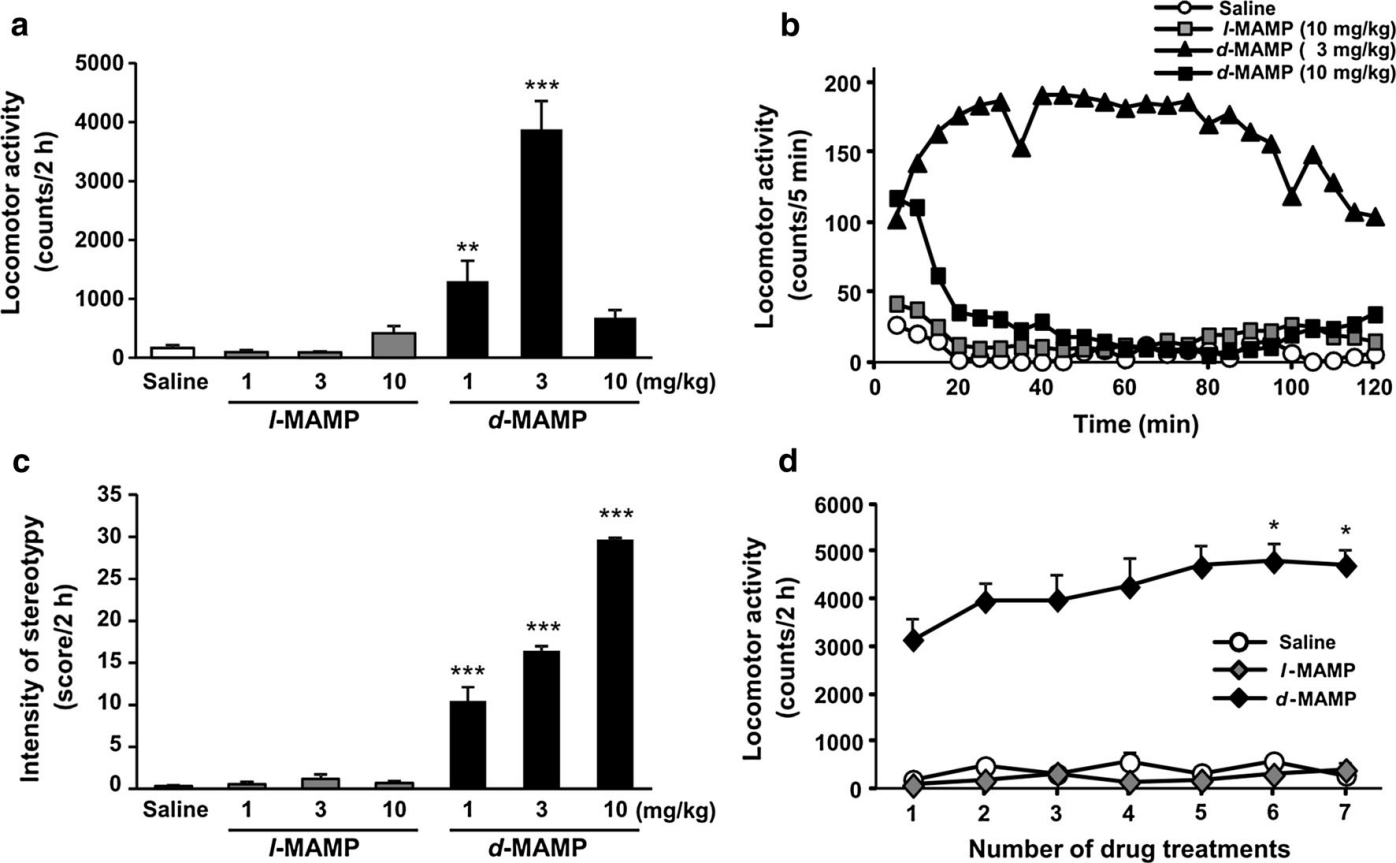
*d*-Methamphetamine, but not *l*-methamphetamine, at doses of 1–10 mg/kg induces psychomotor activity. Cumulative counts (**a**) and temporal change (**b**) in locomotor activity in mice for 2 h following a single administration of saline, *l*-methamphetamine (*l*-MAMP), or *d*-methamphetamine (*d*-MAMP) at doses of 1–10 mg/kg. **c** Cumulative 2-h scores for stereotyped behaviors in mice treated with *l*- or *d*-methamphetamine (1–10 mg/kg). **d** Sensitization following repeated administration of *l*- or *d*-methamphetamine (2 mg/kg). Each value represents mean ± SEM (**a**, **c**, and **d**) or mean (**b**). (**a** and **b**, *n* = 12; **c**, *n* = 9; **d**, *n* = 7–8). *P < 0.05, **P < 0.005 and ***P < 0.0005 vs. saline-treated mice (**a**, **c**), or vs. the first administration in each group (**d**)

Subcutaneous administration of *l*-methamphetamine at doses of 1–10 mg/kg did not significantly increase locomotor activity in mice (Fig. 1a). By contrast, administration of *d*-methamphetamine at doses of 1–3 mg/kg led to dose-dependent increases in locomotor activity. Although marked increases in locomotor activity were measured during the first 10 min following administration of *d*-methamphetamine at 10 mg/kg, this dose did not significantly augment cumulative locomotor activity during the entire 2-h period (Fig. 1b). However, *d*-methamphetamine-treated mice showed intense stereotyped behaviors (e.g., biting or licking) without traveling, even beyond the 2-h period. The stereotyped behaviors were evaluated at the same doses. *d*-Methamphetamine induced stereotyped behaviors in a dose-dependent manner, whereas *l*-methamphetamine did not (Fig. 1c). This result suggests that the decreased locomotor activity in mice treated with 10 mg/kg of *d*-methamphetamine may be due to the induction of strong stereotyped behaviors. Moreover, mice repeatedly administered *l*-methamphetamine did not develop behavioral sensitization, whereas repeated exposure to *d*-methamphetamine led to hyperlocomotion at a level exceeding that induced following the initial administration (Fig. 1d).

### Pharmacokinetics

We next investigated whether differences in plasma or brain pharmacokinetic parameters reflected the intensity of the psychomotor effects. Values of plasma *C*_max_ and AUC_0–4h_ following administration of 1 mg/kg *d*-methamphetamine were comparable with those for 1 mg/kg *l*-methamphetamine (Table 1). Mice were administered 1 mg/kg of *d*-methamphetamine s.c. (a dose that induced psychomotor activity) or 10 mg/kg (s.c.) of *l*-methamphetamine (the maximum dose used in the behavioral tests). Plasma *C*_max_, AUC_0–2h_, and striatal concentrations of methamphetamine and amphetamine following administration of *l*-methamphetamine were ≥10-fold those post *d*-methamphetamine administration. These results indicate that the distinctive psychomotor effects of *d*- and *l*-methamphetamine are not due to differences in their plasma or striatum pharmacokinetics.

**Table 1:** Pharmacokinetic parameters and brain concentrations of methamphetamine and amphetamine in mice following subcutaneous administration of *l*-methamphetamine or *d*-methamphetamine

Exp. no.	Tissue	Analyte	Parameter	Drug administered	
				*l*-MAMP (1 mg/kg)	*d*-MAMP (1 mg/kg)
I	Plasma	MAMP	*C*_max_ (μg/mL)	0.062 ± 0.007	0.072 ± 0.013
			AUC_0–4 h_ (μg·h/mL)	0.129	0.159
				*l*-MAMP (10 mg/kg)	*d*-MAMP (1 mg/kg)
II	Plasma	MAMP	*C*_max_ (μg/mL)	0.988 ± 0.034*	0.093 ± 0.008
			AUC_0–2 h_ (μg·h/mL)	1.66 ± 0.06*	0.142 ± 0.008
		AMP	*C*_max_ (μg/mL)	0.067 ± 0.005	<0.003^a^
			AUC_0–2 h_ (μg·h/mL)	0.092 ± 0.008	N.C.
	Brain	MAMP	Conc. (μg/g tissue)	1.99 ± 0.06*	0.126 ± 0.008
		AMP	Conc. (μg/g tissue)	0.212 ± 0.013*	0.006 ± 0.001

Blood samples were collected at 0.08, 0.17, 0.33, 0.5, 1, 1.5, 2, 3 and 4 h (Exp. I), and 0.17, 0.33, 0.5, 1, 1.5, and 2 h (Exp. II) post-drug administration. Each value represents mean or mean ± SD (3–4 mice per time-point; Exp. I), or mean ± SEM (6 mice per group; Exp. II)

*MAMP* methamphetamine, *AMP* amphetamine, *AUC*_*0–2h*_ *and AUC*_*0–4h*_ area under the plasma concentration vs. time curve from 0 to 2 or 4 h, *C*_*max*_ maximum plasma concentration, *Conc.* concentration, *N.C.* not calculated

* *P* < 0.05 vs. *d*-methamphetamine-treated group

^a^Below the lower limit of quantitation (3 ng/mL)

## Discussion

There have been no studies directly comparing the pharmacodynamics and pharmacokinetics of the methamphetamine enantiomers in mice. It is often suggested that *d*-methamphetamine exerts more potent physiological and pharmacological effects than *l*-methamphetamine does, and that the stimulating effects exerted by *l*-methamphetamine on the central nervous system are 2–10 times less potent than those of *d*-methamphetamine (Mendelson et al. 2006). The results of the present study indicated that psychostimulant effects induced by *l*-methamphetamine are lower than those elicited by one-tenth the dose of *d*-methamphetamine. In addition, plasma pharmacokinetic parameters and striatal concentrations of methamphetamine following administration of *l*-methamphetamine at 10 mg/kg (which did not induce psychomotor activity) were approximately 11 and 16 times as high, respectively, as those following administration of 1 mg/kg *d*-methamphetamine. Despite the fact that there are differentiable psycho-stimulating effects between two enantiomers, no significant difference in plasma pharmacokinetic parameters was detected at 1 mg/kg. In comparative positron emission tomography studies, the pharmacokinetics in the baboon brain was comparable for ^11^C-*d*- and ^11^C-*l*-methamphetamine (Fowler et al. 2007). Thus, factors other than brain or plasma pharmacokinetics, especially differences in the affinity of each enantiomer for its pharmacological targets, may account for the more potent psychomotor effects of *d*-methamphetamine. For instance, the effects of *d*-methamphetamine on the release and uptake of dopamine in rat caudate synaptosomes are reportedly approximately 17- and 42-fold greater, respectively, than those of *l*-methamphetamine (Rothman et al. 2001). Kuzcenski et al. (1995) demonstrated that the peak dopamine concentration in rat caudate following s.c. administration of 2 mg/kg *d*-methamphetamine is approximately 2.3 times as high as that after administration of 12 mg/kg *l*-methamphetamine. Comparative studies to differentiate the affinities of the enantiomers to target molecules will be required to clarify the mechanisms that give rise to the difference in psychomotor efficacies between *d*- and *l*-methamphetamine.

Selegiline is sometimes regarded as an inducer of psychoactive effects through its metabolites having a component of *N*,α-dimethyl-*N*-2-propynyl phenethylamine. Previous clinical studies have reported that the *C*_max_ of *l*-methamphetamine following administration of conventional selegiline tablets 10 mg (Clarke et al. 2003) was fivefold lower than the *C*_max_ observed in methamphetamine abusers who had received intravenous *l*-methamphetamine at a dose of 0.25 mg/kg, which does not exert psychoactive effects (Mendelson et al. 2006). Thus, the results of these previous reports suggest that the *l*-methamphetamine available as a metabolite after selegiline administration at clinical doses may have little potential to induce psychoactive effects.

Taken together, our results indicated that the psychostimulant effects elicited by *d*-methamphetamine are at least 10 times stronger than those induced by *l*-methamphetamine based on their doses for inducing psychomotor activities. Furthermore, the distinct psychoactive efficacies of the enantiomers are not due to differences in plasma pharmacokinetics or brain concentrations of methamphetamine/amphetamine following administration of the respective enantiomers.
